# Rapid Increase in Soil Respiration and Reduction in Soil Nitrate Availability Following CO_2_ Enrichment in a Mature Oak Forest

**DOI:** 10.1021/acsomega.4c09495

**Published:** 2025-01-02

**Authors:** Angeliki Kourmouli, R. Liz Hamilton, Johanna Pihlblad, Rebecca Bartlett, Angus Robert MacKenzie, Iain P. Hartley, Sami Ullah, Zongbo Shi

**Affiliations:** † Birmingham Institute of Forest Research (BIFoR), University of Birmingham, Edgbaston B15 2TT, U.K.; ‡ Lancaster Environment Centre, Lancaster University, Bailrigg LA1 4YQ, U.K.; § School of Geography, Earth & Environmental Sciences, University of Birmingham, Edgbaston B15 2TT, U.K.; ∥ Geography, Faculty of Environment, Science and Economy, University of Exeter, Exeter EX4 4RJ, U.K.

## Abstract

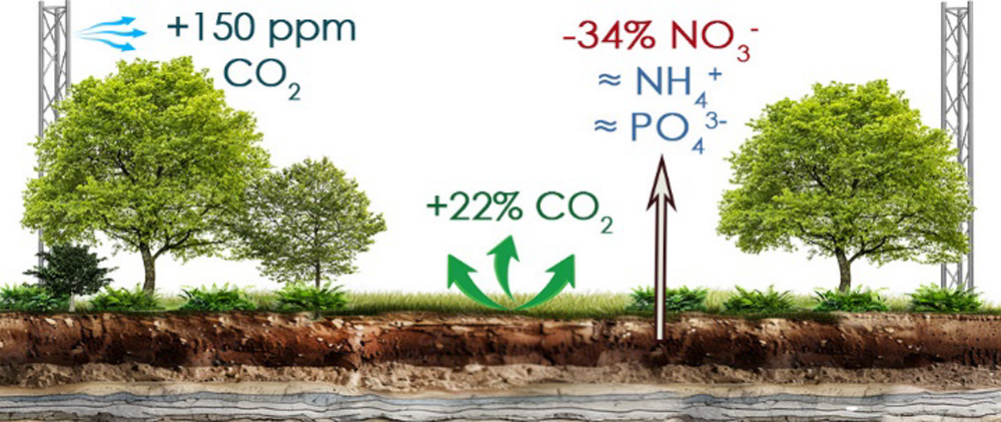

In the future, with elevated atmospheric CO_2_ (eCO_2_), forests are expected to increase woody biomass to capture more carbon (C), though this is dependent on soil nutrient availability. While young forests may access unused nutrients by growing into an unexplored soil environment, it is unclear how or if mature forests can adapt belowground under eCO_2_. Soil respiration (*R*_s_) and nutrient bioavailability are integrative ecosystem measures of below-ground dynamics. At Birmingham’s Institute of Forest Research Free Air CO_2_ Enrichment (BIFoR FACE) facility, we investigated the effects of eCO_2_ (+150 ppm above ambient) on a mature oak forest during the first year of exposure. We observed an annual R_s_ increase of ∼21.5%; 996 ± 398 g C m^–2^ year^–1^ (ambient) to 1210 ± 483 g C m^–2^ year^–1^ (eCO_2_). The eCO_2_ impact was greater on belowground nutrient cycling, with monthly nitrate availability decreasing by up to 36%. These results show that high C uptake resulted in higher soil respiration with a concomitant decrease in the level of soil nitrate during the first year. These belowground responses and their long-term dynamics will have implications for the carbon budget of mature forest ecosystems in changing climate.

## 1 Introduction

Forests predominantly serve as carbon (C) sinks, sequestering approximately one-third (∼0.7 Pg C year^–1^) of the annual C uptake by terrestrial ecosystems.^1−3^ The C sink’s magnitude depends on the balance between photosynthetic inputs and carbon dioxide (CO_2_) release via soil respiration (*R*_s_), including that from plant roots, soil microbes, hyphae, and soil fauna.^4^ Theoretically, mature forests in stable environments can approach equilibrium regarding their growth/turnover and litter inputs/*R*_s_ rates, resulting in near-zero net fluxes and a comparatively constant C storage.^5^ However, elevated (eCO_2_) can cause this equilibrium to shift, either increasing or decreasing the C storage capacity of the ecosystem. eCO_2_ has been shown to enhance the uptake of CO_2_ into plant and soil biomass^1^ and increase gross primary productivity (GPP),^6^ but confounding factors, including higher temperatures, moisture limitation, and progressive nutrient limitation, may offset or constrain C gains.^7^

The C storage capacity of newly planted and secondary forests has been studied extensively^8^; however, mature temperate forests are considered to store over twice as much C per unit area than their younger counterparts.^9^ Mature forests may have thoroughly explored and exploited their belowground resources, exhibiting more tightly coupled nutrient cycles than young forests,^10^ thereby limiting the possibility of increased C allocation belowground.^11^ Mature trees may also have less flexibility in nutrient use,^12^ potentially reducing their impact on soil nutrient availability under eCO_2_. Accordingly, small perturbations to the net C balance in mature forests may have substantial implications for the global C cycle.^13^

Although mature forests are crucial for global C dynamics,^14^ our understanding of their response to climate change remains incomplete. Warmer climate with a higher frequency of wet/dry cycles may enhance *R*_s_ through increased microbial activity and decomposition rates,^15^ eCO_2_ may stimulate plant growth and promote C sequestration through increased organic matter inputs to soil or stimulate decomposition of soil organic matter and repress C sequestration through increased root exudation.^16^ Uncertainty remains about how mature temperate forests respond to changes in nutrient availability, as the magnitude and persistence of eCO_2_-driven plant growth depend on nitrogen (N) and phosphorus (P) availability.^17−20^ Thus, mature forests account for a significant source of uncertainty when assessing the potential feedback in global environmental change.

Additional N and P are required to maintain forest growth under eCO_2_ in forests severely limited by nutrient availability. Greater biomass accumulation has been observed under eCO_2_ in young stands or relatively homogeneous plantations,^21−23^ as well as, in mature forests.^24^ In contrast to the substantial attention given to the effects of eCO_2_ on the N cycle, impacts of eCO_2_ on P availability, or combined N and P availability, have received less attention. The impacts of eCO_2_ on both N and P availability, without nutrient fertilization, have only been studied at a mature P-limited *Eucalyptus* forest where increases in the availability of both nutrients were observed.^25−27^ Thus, monitoring short-term responses of belowground nutrient dynamics can be crucial for the potential of mature forest ecosystems’ C-accumulation under eCO_2_.

Early indications at BIFoR FACE suggest eCO_2_ increased photosynthetic capacity,^28^ fine root net primary productivity,^29^ woody biomass,^24^ and decreased nitrification.^30^ The present study investigated high-frequency *R*_s_ measurements 5 months prior to, and during the first year of, the CO_2_ enrichment, while simultaneously measuring the bioavailability of ammonium (NH_4_^+^-N), nitrate (NO_3_^–^-N), and phosphate (PO_4_^3–^-P) at monthly intervals. We hypothesize that the observed increase in photosynthetic uptake^28^ and fine root net primary productivity^29^ will drive higher *R*_s_ under eCO_2_.

## 2 Materials and Methods

### 2.1 Experimental Site

The University of Birmingham’s Institute of Forest Research (BIFoR) FACE forest is a temperate deciduous woodland located in Staffordshire, England (52° 48′ 3.6” N, 2° 18′ 0” W). Total annual precipitation for the site measured in 2016 and 2017 was 871 and 713 mm, respectively, and the mean annual temperature was 11.6 and 10.3 °C, respectively. The soil is dystric cambisol with a sandy loam with 12% clay and 15% silt texture (inceptisol) and a mean soil pH of 4.5, C of 3.95 ± 1.56% (mean ± sd) and N 0.27 ± 0.09% (mean ± sd) in the top 10 cm.^31^ The woodland has a dense multilayered canopy that was undisturbed during the construction of the FACE infrastructure and a detailed site description can be found at refs (32,33). The dominant overstorey species is old-growth (age >160 years) *Quercus robur* (pedunculate oak), approximately 25 m in height with a leaf area index of ∼6.^24^ The understorey is composed of *Corylus avellana* (common hazel) coppice, interspersed with self-set *Acer pseudoplatanus* (sycamore), *Ilex aquifolium* (holly), and *Crataegus monogyna*, (hawthorn). There is a sparse ground flora, mostly of bramble (*Rubus fruticosus* agg.), bluebell (*Hyacinthoides nonscripta*; see ref (34)), fern (*Dryopteris* sp.), and honeysuckle (*Lonicera periclymenum*).

The *in-situ* response of mature forest systems to climate change factors requires an ecosystem-scale experimental design to incorporate tall canopy trees under realistic growth conditions and complex biological interactions. The emerging network of next-generation free-air carbon dioxide enrichment (FACE) experiments that include BIFoR FACE (temperate oak forest), EucFACE (mature Eucalyptus stand in Australia), and AmazonFACE (primary rainforest) seek to expand our understanding of mature forest ecosystem response to eCO_2_ across different biomes.^35^

The FACE facility comprises six 30-m diameter infrastructure arrays, three of which receive eCO_2_ at +150 ppm (eCO_2_, hereafter) above ambient and three of which receive ambient CO_2_ (i.e., forest air piped into the array using infrastructure replicating the treatment arrays; aCO_2_, hereafter;^32^), representing the predicted atmospheric CO_2_ levels in 2050.^36^ The array selection was determined after baseline assessments of several forest characteristics and conducted during the planning stages of the experiment.^32^ The first year of CO_2_ enrichment was applied from oak budburst to leaf fall, which for the study period was 3 April and was maintained 27 October 2017 (*Year 1*, hereafter) and took place during daylight hours (see Supporting Information). During the CO_2_ enrichment, eCO_2_ arrays were within 10% of the target (i.e., ∼15 ppm) for 81.6% of the scheduled operation time and 20% of the target within 96.7% of the scheduled operation time, with average Year 1 concentration of 400 ± 17 versus 547 ± 21 (mean ± sd) ppm for aCO_2_ and eCO_2_ arrays, respectively.^32^

### 2.2 Belowground Respiration and Nutrient Measurements

#### 2.2.1 Soil Respiration, *R*_s_

In spring 2016, pseudoreplication of three PVC (Drainage Superstore, CMO Ltd., Plymouth, UK) collars was randomly placed and permanently situated within each of the six infrastructure arrays (*n* = 3 eCO_2_ arrays; *n* = 3 aCO_2_ arrays), ∼2 m from the closest oak tree, to monitor the *R*_s_. Collars were sealed to the soil surface, to avoid severing roots, and left over during the growing season to minimize disturbance until the first measurements began in autumn 2016. Hourly measurements of *R*_s_ were made from 19 October 2016 (5 months before the beginning of CO_2_ enrichment, *Pre-treatment,* hereafter) to 31 December 2017 (2 months after the switch-off of CO_2_ enrichment at the end of the first growing season) using 6 long-term automatic chambers interfaced with a multiplexer and an infrared gas analyzer (IRGA) (Li-8100–104 long-term chambers, Li-8150 multiplexer, and Li-8100A IRGA LI-COR). The distance between arrays necessitated measurements in two arrays (an eCO_2_ array and its aCO_2_ pair; pairings were established based on baseline studies determining several ecosystem characteristics) at any one time period. Each array pair was measured by rotating the chambers every 2 weeks between the three paired eCO_2_ and aCO_2_ arrays on a 6-week rotation. Details for the measurement cycle can be found in the Supporting Information.

All data went through quality checks before calculating the linear regressions of CO_2_ flux using SoilFluxPro software, version 4.0.1 (LI-COR, Nebraska, USA) (Supporting Information). Data with poor quality fits, including negative linear fluxes and those linear fluxes derived from observations when the coefficient of variation is higher than 3.5, were removed. Of 69,216 data points during the first year of operation, 39,557 (57%) data points were used for analysis, primarily due to equipment failures (e.g., water condensation in the electric plates, pump failures, and power cuts) and, to a lesser extent, due to poor-quality regression fits. However, over 90 and 84% of the measurement days experienced less than 20% data loss for aCO_2_ and eCO_2_ arrays, respectively (see the Supporting Information). Daily and monthly averages of these data are reported in this study in μmol of CO_2_ m^–2^ s^–1^; annual averages are reported in g C m^–2^ year^–1^.

#### 2.2.2 Environmental Drivers

Soil volumetric water content (VWC) and soil temperature (*T*_s_) were measured with permanently installed shallow CS655 probes inserted in the top 12 cm of soil (Campbell Scientific, Logan, UT, USA). VWC probes were sited one m from each of the three experimental *R*_s_ collars in each array. The VWC data were recorded in 15 min intervals by a data logger in each plot (CR1000, Campbell Scientific) and are reported as daily averages. The precipitation is reported as a daily sum and was measured by ARG100 rain gauges (Campbell Scientific, Logan, UT, USA) sited above and outside the forest.^33^

#### 2.2.3 Bioavailable Inorganic N and P

Ion-exchange resin membranes (Membranes International Inc., New Jersey, USA) were used to measure the cumulative bioavailable inorganic N, as nitrate (NO_3_^–^-N), ammonium (NH_4_^+^-N) and P, as phosphate (PO_4_^3–^-P). Between May and October 2016, five anion-exchange membranes (AMI 7001) and five cation-exchange membranes (CMI 7000) were inserted in the top 10 cm of the soil, ∼50 cm of the *R*_s_ collars, in each array and analyzed individually (for preconditioning of the ion-exchange resin membranes, see the Supporting Information). Following a statistical analysis of replicate variability (data not shown), the sampling design was modified to increase the statistical power. From January 2017, triplicate anion- and cation-exchange resin membranes were inserted in the soil at eight locations within each array; three sets were located next to each of the *R*_s_ collars, and the other five sets were randomly located within each array. Membranes were deployed in situ for approximately one month before retrieval for a cumulative assessment of nutrient availability (for resin membranes loading capacity evaluation see Supporting Information). Cumulative monthly *R*_s_ was calculated for the deployment period of the ion exchange membranes. These cumulative values were then used to correlate *R*_s_ with available NO_3_^–^-N, NH_4_^+^-N, and PO_4_^3–^-P.

Membranes were extracted for NO_3_^–^-N, NH_4_^+^-N, and PO_4_^3–^-P using 0.5 M HCl after shaking at 180 rpm for 2 h.^37,38^ From January 2017, the triplicate membranes from each location were bulked to give eight samples per array. Concentrations of NO_3_^–^-N and NH_4_^+^-N were analyzed by a continuous flow analyzer (San++ Continuous Flow Analyzer, Skalar, Breda, The Netherlands); concentrations of PO_4_^3–^-P were analyzed by ultraviolet–visible spectroscopy (Jenway 6850) using the molybdenum blue method.^39^ Limits of detection and limits of quantitation are given in the Supporting Information*.* The data presented in this study are reported as monthly averages with a standard deviation, per unit area of the membrane, in μg NH_4_^+^-N cm^–2^ day^–1^, μg NO_3_^–^-N cm^–2^ day^–1^, and μg PO_4_^3–^-P cm^–2^ day^–1^, respectively.

Response ratios (RR) for all three soil bioavailable nutrients were calculated using the approach for two-group experimental design studies^40^:
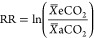
1where RR is the natural log proportional change in the means (*X̅*) of the nutrient availabilities in eCO_2_ and aCO_2_ arrays. The associated sampling variance was calculated as
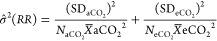
2where SD is the standard deviation, and *N* is the sample size of *X̅*aCO_2_ and *X̅*eCO_2_, respectively.

### 2.3 Statistical Analysis

Data analyses were carried out in R 3.6.2 and RStudio 1.2.5033.^41^ Mixed-effects models are a commonly used method when handling complex ecological data sets with inherently high variability and multiple sources of variation (e.g., see ref (42)). Mixed-effects models were performed using the R package *lme4*,^43^ and the coefficient of determination (*R*^2^) was calculated using the R package *MuMIn*.^44^ For all analyses, effects significant at the 95% level (i.e., model-reported *p* < 0.05) were considered conclusive, while those with 0.05 < *p* < 0.15 were considered indicative. For CO_2_ treatment, the number of days since the beginning of the experiment (*Time*, hereafter), as well as the interactions with the environmental drivers (VWC and *T*_s_) were treated as fixed effects for both *R*_s_ and bioavailable soil nutrients models. In all mixed-effects models performed, the location of the chamber was nested within the experimental ‘array’, and a random slope and intercept model was applied. Time as the number of days since the beginning of the experiment was also added as a random effect to account for the correlations between repeated measurements within each array. The significance of the fixed effects and their interactions were evaluated using the Anova function in the *Car* package^45^ with the Kenward-Rodger approximation for estimating the degrees of freedom. All models were evaluated for homoscedasticity, normality, linearity of residuals, and linearity of the random effects (for further information, see Supporting Information).

#### 2.3.1 Estimation of Annual *R*_s_

Due to the experimental setup (measuring sequentially each aCO_2_ array and its paired eCO_2_ array for 2 weeks) and the seasonal and temporal variability of *R*_s_, annual *R*_s_ was estimated from the best-fitted linear model of *T*_s_ and VWC for aCO_2_ and eCO_2_ arrays separately splitting the available data into training and testing data sets (20 to 80% split) and evaluating the model performance based on lowest Root Mean Square Error (RMSE), lowest Akaike Information Criterion (AIC,^46^), and the highest adjusted *R*^2^ (see the Supporting Information). Hourly averages of the *R*_s_ data were then predicted for the year using the best model per CO_2_ treatment on independent averages of the *T*_s_ and VWC data. The annual *R*_s_ values were then calculated as the sum of the predicted *R*_s_ rate for the year.

## 3 Results and Discussion

### 3.1 *R*_s_ Response to ECO_2_ and Estimation of Annual *R*_s_

**Figure 1:**
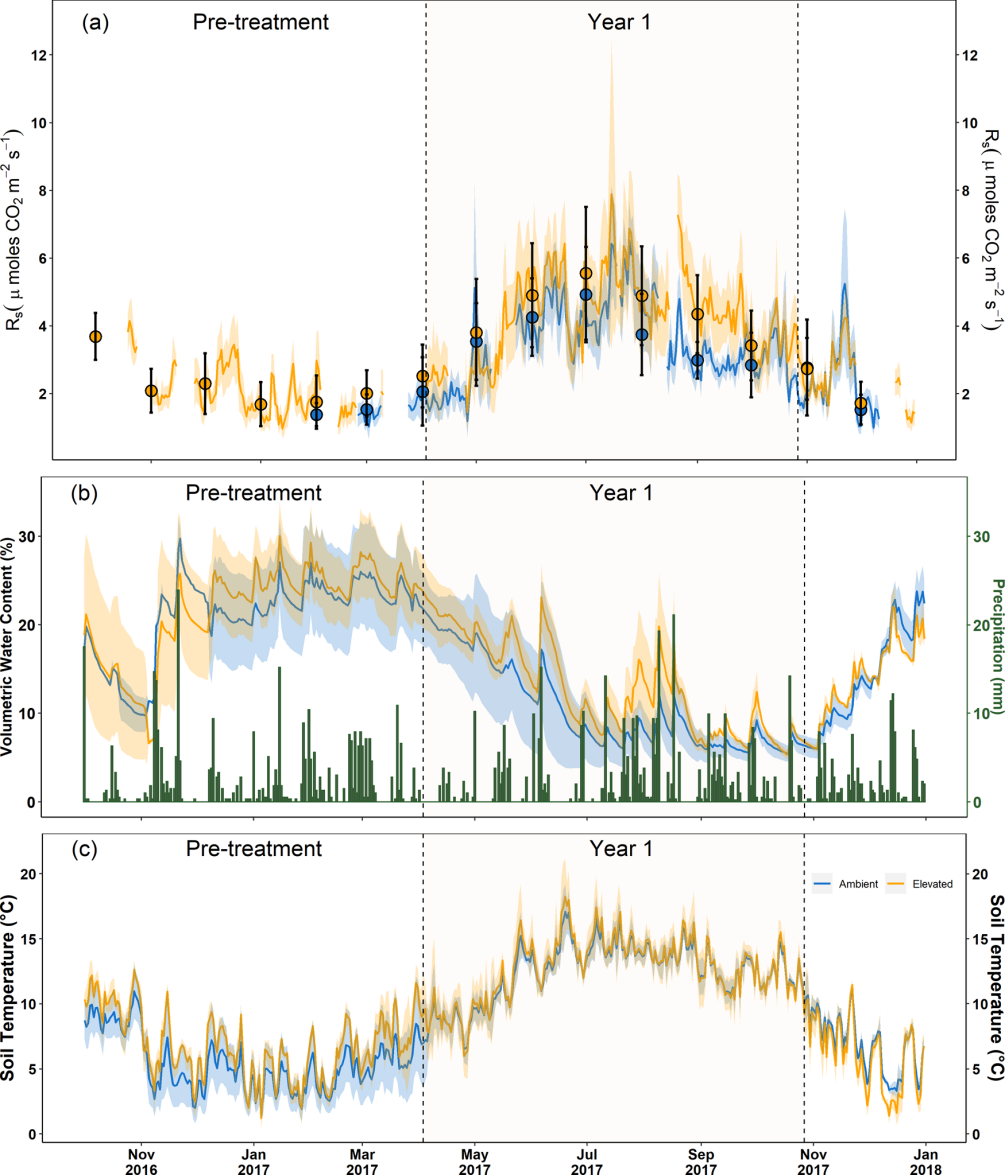
Seasonal variation of *R*_s_ (a), VWC and precipitation (b), and *T*_s_ (c) at BIFoR FACE during the Pre-treatment and Year 1 of CO_2_ enrichment (October 2016–December 2017). The yellow colored lines/solid points denote the eCO_2_arrays, while the blue lines/solid points denote the aCO_2_ arrays. The lighter colored ribbons around the lines and the error bars indicate the standard deviation for *R*_s_, VWC, and *T*_s_. Lines denote daily averages and points monthly averages (at the start of each calendar month). Error bars show the standard deviation. The vertical dashed lines enclose the period when the CO_2_ enrichment was applied (Year 1, 3rd April–27th October 2017).

*R*_s_ was highly seasonal in both Pre-treatment and Year 1 and followed seasonal patterns related to VWC, *T*_s_, and precipitation (Figure 1). Precipitation was sporadic and highly variable (Figure 1b), leading to a pulse effect on *R*_s_ co-occurring with an increase in VWC. Daily and monthly mean *R*_s_ were marginally higher (13.3%) in eCO_2_ during the Pre-treatment (1.5 ± 0.4sd μmol CO_2_ m^–2^ s^–1^ in aCO_2_ versus 1.7 ± 0.7sd μmol CO_2_ m^–2^ s^–1^ in eCO_2_). However, no statistically significant differences were observed between arrays preassigned as “aCO_2_” and “eCO_2_” for the CO_2_ treatment alone (*p* = 0.225, Table 1) or when combined with either *T*_s_ or VWC alone. A significant difference was observed for the effect of time (*p* = 0.049) and when combined with both *T*_s_ and VWC (*p* = 0.037). However, neither model was a good predictor of the observed *R*_s_, with the majority of the variability being explained by the random effects of location and day of the year.

**Table 1:** Summary of mixed-effects models of environmental drivers and CO_2_ treatment on daily *R*_s_ during Pre-treatment and Year 1 at BIFoR FACE^a^

	Pre-treatment				Year 1			
Model	*p*	*R*^2^_m_	*R*^2^_c_	AIC	*p*	*R*^2^_m_	*R*^2^_c_	AIC
*R*_*s*_ = β_0_ + CO_2_ + γ + ε	0.225	0.109	0.928	520	0.377	0.033	0.832	2955
*R*_*s*_ = β_0_ + CO_2_ × Time + γ + ε	**0.049**	0.169	0.904	527	**8.71 × 10**^**–6**^	0.059	0.838	2952
*R*_*s*_ = β_0_ + CO_2_ × *T*_s_ + γ + ε	0.374	0.184	0.900	465	**0.001**	0.289	0.820	2724
*R*_*s*_ = β_0_ + CO_2_ × VWC + γ + ε	0.201	0.121	0.927	508	**3.52 × 10**^**–2**^	0.051	0.842	2880
*R*_*s*_ = β_0_ + CO_2_ × *T*_s_ × VWC + γ + ε	**0.037**	0.238	0.902	501	**1.73 × 10**^**–5**^	0.309	0.830	2753
*R*_*s*_ = β_0_ + CO_2_ × Time × *T*_s_ + γ + ε	0.707	0.417	0.853	465	0.525	0.315	0.832	2742
*R*_*s*_ = β_0_ + CO_2_ × Time × VWC + γ + ε	0.446	0.379	0.897	512	**0.011**	0.070	0.844	2925
*R*_*s*_ = β_0_ + CO_2_ × Time × *T*_s_ × VWC + γ + ε	0.618	0.48	0.907	559	0.797	0.335	0.842	2831

a Fixed factors are CO_2_ treatment, time, VWC, and *T*_s_ and their interactions where *y* represents the random slope and intercept of plot location nested within the array and the random intercept of time. *p* < 0.05 is shown in bold, otherwise not significant. The variables’ coefficients of determination describing models’ predictive capacity including both fixed effects only (*R*^2^_m_) and fixed and random effects (*R*^2^_c_) together with Akaike Information Criteria (AIC) are also shown.

By contrast, mean *R*_s_ in eCO_2_ was ∼223% higher than that in aCO_2_ through Year 1 (3.5 ± 1.3sd vs 4.3 ± 1.7sd μmol CO_2_ m^–2^ s^–1^ for aCO_2_ and eCO_2_, respectively; Table 2). While CO_2_ treatment alone did not have a significant effect on *R*_s_ in Year 1 (*p* = 0.377), there was a clear divergence in mean monthly respiration over the period of CO_2_ enrichment reflected in the significant interaction of CO_2_ treatment with Time (*p* = 8.71 × 10^–6^). The period of peak plant activity between June and October showed the greatest differences in mean *R*_s_ between aCO_2_ and eCO_2_. Notably, significant differences in mean monthly *R*_s_ between aCO_2_ and eCO_2_ were not apparent for the periods before bud burst and after leaf senescence (i.e., May or November) but were apparent for each month in between (i.e., June to October;Table 2). The greatest monthly mean *R*_s_ differences were ∼32% (3.7 ± 1.1 (mean ± sd) μmol of CO_2_ m^–2^ s^–1^ in aCO_2_ compared to 4.9 ± 1.4 (mean ± sd) μmol of CO_2_ m^–2^ s^–1^ in eCO_2_) and 43% (3.0 ± 0.5 μmol of CO_2_ m^–2^ s^–1^ in aCO_2_ compared to 4.3 ± 1.0 μmol of CO_2_ m^–2^ s^–1^ in eCO_2_) observed in August and September, respectively. *R*_s_, VWC, and *T*_s_ were all higher in eCO_2_ than in aCO_2_ (Figure 1) which resulted in a highly significant interaction effect of VWC and *T*_s_ (*p* = 1.73 × 10^–5^) that explained 31% of the variability in observed *R*_s_ (Table 1).

**Table 2:** Mean monthly *R*_s_ (sd in parentheses) in aCO_2_ and eCO_2_ arrays for February 2016 to November 2017^a^

	Mean monthly *R*_s_ (μmol CO_2_m^–2^ s^–1^)			
	month	aCO_2_	eCO_2_	*p*
Pre-treatment	February	1.38 (0.27)	1.68 (0.70)	0.217
	March	1.53 (0.35)	2.01 (0.57)	**6.3 × 10^–7^**
Year 1	April	2.01 (1.04)	2.45 (0.63)	0.9753
	May	3.54 (0.88)	3.78 (1.42)	0.975
	June	4.24 (0.99)	4.84 (1.32)	**4.0 × 10**^**–4**^
	July	4.95 (1.30)	5.82 (2.42)	**0.004**
	August	3.74 (1.12)	4.85 (1.37)	**2.2 × 10**^**–7**^
	September	2.99 (0.46)	4.31 (1.00)	**2.2 × 10**^**–16**^
	October	2.87 (0.97)	3.39 (0.91)	**4.4 × 10**^**–5**^
	November	2.76 (1.29)	2.74 (0.81)	0.304

a *p* shows significance for a *t*-test (or non-parametric equivalent) of means between aCO_2_ and eCO_2_ arrays.

**Figure 2:**
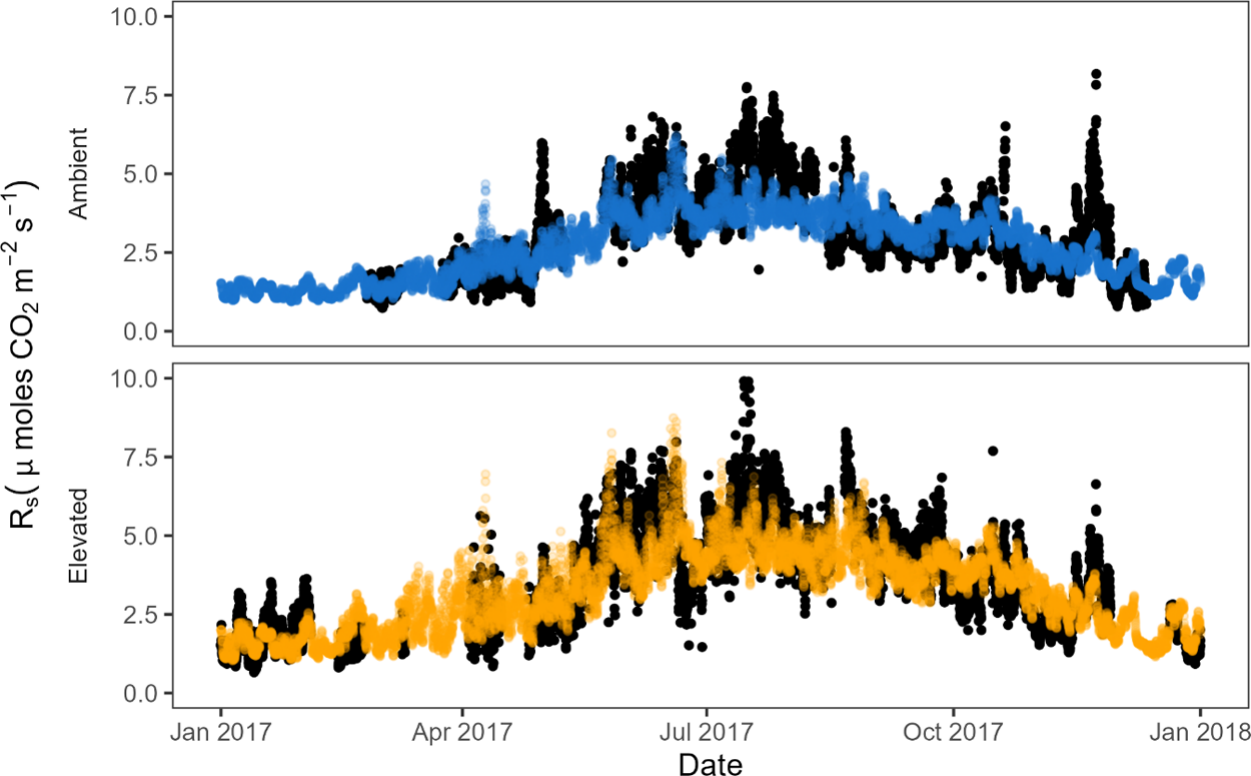
Observed (black points) and predicted *R*_s_ hourly respiration under aCO_2_ (top) and eCO_2_ (bottom) arrays in μmol of CO_2_ m^–2^ s^–1^.

Using the best-fitted models (i.e., the two-way VWC × *T*_s_ interaction) for aCO_2_ and eCO_2_ separately, we could use continuous data of soil moisture and temperature to estimate hourly flux rates and calculate an annual respiration rate for the calendar year (January 01 to December 31). The estimated mean annual *R*_S_ for 2017 was approximately 21.5% higher in eCO_2_ (1210 ± 483 g C m^–2^ year^–1^; mean ± sd) (Figure 2 bottom) compared to aCO_2_ (996 ± 398 g C m^–2^ year^–1^; mean ± sd) (Figure 2 top).

The estimates of aCO_2_ annual *R*_s_ from this study are within the (very broad) range of observations (500–2100 g C m^–2^ year^–1^) reported for similar temperate forests^47^ and are marginally higher than global mean estimates for deciduous broadleaf forests: ∼850 g C m^–2^ year^–1^.^48^ The 21.5% increase in annual *R*_s_ is broadly commensurate with other treatment effects measured in the first years of CO_2_ enrichment at BIFoR FACE. For example,^28^ report a maximum monthly average 33% increase in net photosynthesis under eCO_2_ for the upper oak canopy for June 2017 and an overall average treatment effect of 23 ± 4% for 2017–2019 (Year 1–3 of CO_2_ enrichment). Similarly,^29^ found an 18% increase in fine root net primary productivity (NPP_froot_) for 2017 (Year 1 of CO_2_ enrichment) and^24^ found a 10% increase in dry matter increment over 7 years of CO_2_ enrichment (2017–2023).

Previous FACE experiments in forest ecosystems have observed different magnitudes of increase (Table 3) in *R*_s_ during their first year of CO_2_ enrichment. This variability in response remains when normalized against the percentage increase in the size of the CO_2_ treatment across different experiments (Table 3, column 5). Species, plant age, and experimental setup also vary in Table 3, making a general pattern impossible to discern. Of the two prior studies on mature forests, results from this study are similar in magnitude to those reported for WebFACE,^12^ and to EucFACE in respect of having roughly equal treatment effects in C assimilation and *R*_s_.^49^ The observed increase during the first year of CO_2_ enrichment in annual *R*_s_ at EucFACE (the only other study for mature forest) was approximately 7–11%^50,51^; lower than the observed increase at BIFoR FACE (∼21.5%). These two systems vary greatly in their soil pedogenesis, which influences their soil types, processes, and nutrient cycling, but they also differ in the number of tree species. An ecosystem with higher plant diversity, such as BIFoR, will potentially have a higher variability in its response to eCO_2_. This can potentially explain the difference in the magnitude of the eCO_2_ effect in *R*_s_ between the two sites, meaning that mixed-stand forests exposed to eCO_2_, although mature in principle, could have a higher impact on *R*_s_.

**Table 3:** Forest Free Air CO_2_ Enrichment (FACE) experiments and the CO_2_ treatment effect in *R*_s_ during their first year of CO_2_ enrichment, in descending chronological order^a^

Site	Applied CO_2_ treatment (eCO_2_/aCO_2_, %)	Species	*R*_s_ treatment effect (%)	*R*_s_ treatment effect/CO_2_ treatment	References
Duke FACE–FACTS I	52	*Pinus taeda*	29	0.6	(King et al.,^21^; U.S. DOE, 2020)
ORNL	38	*Liquidambar styraciflua*	8	0.2	(King et al.,^21^; U.S. DOE, 2020)
Aspen FACE–FACTS II	58	*Populus tremuloides*	13	0.2	(King et al.,^21^; U.S. DOE, 2020)
		*Betula papyrifera*	43	0.7	
POPFACE^a^	48	*Populus alba*	36	0.8	(King et al.,^21^; U.S. DOE, 2020)
		*Populus nigra*	50	1.0	
		*Populus europea*	36	0.8	
WebFACE^a^	63	*Fagus sylvatica*	25^1^	0.4	(Körner et al.,^12^)
		*Quercus petraea*			
EucFACE	38	*Eucalyptus tereticornis*	10	0.3	(Drake et al.,^50^)
BIFoR FACE	38	*Quercus robur* and others	21.5	0.6	this study

a Ambient CO_2_ not provided; the fractional CO_2_ treatment is calculated from the global ambient average for the relevant year.

The observed increase in *R*_s_ in response to eCO_2_ (Table 3, Figures 1a and 2d) likely derives from additional (or qualitatively changed) C inputs, increased microbial activity, increased root respiration, and/or enhanced root growth,^29^ thus leading to a stimulated loss of C from existing soil pools via enhanced decomposition of soil organic matter for nutrient acquisition.^52^ Alterations in the quantity and quality of C inputs to soils, such as litterfall, can explain *R*_s_ responses to eCO_2_.^53^ However, our response during the first year of CO_2_ enrichment cannot be explained by increased litterfall inputs in eCO_2,_ and indeed, no such increase in litterfall was observed (data not shown). The observed increases in photosynthesis,^28^ fine root net primary productivity,^29^ gross N mineralization,^54^ higher leucine amino peptidase activity with higher net N mineralization in the rhizosphere,^30^ and the seasonal variation in the treatment effect, suggest that the positive *R*_s_ response to eCO_2_ was primarily due to increased C allocation belowground to roots and mycorrhizae, which could potentially also prime microbial activity and decomposition of soil organic matter. Nevertheless, we cannot rule out the loss of C from a previously stable soil organic matter pool via nutrient mining.^55,56^

### 3.2 Bioavailable Inorganic N and P

**Figure 3:**
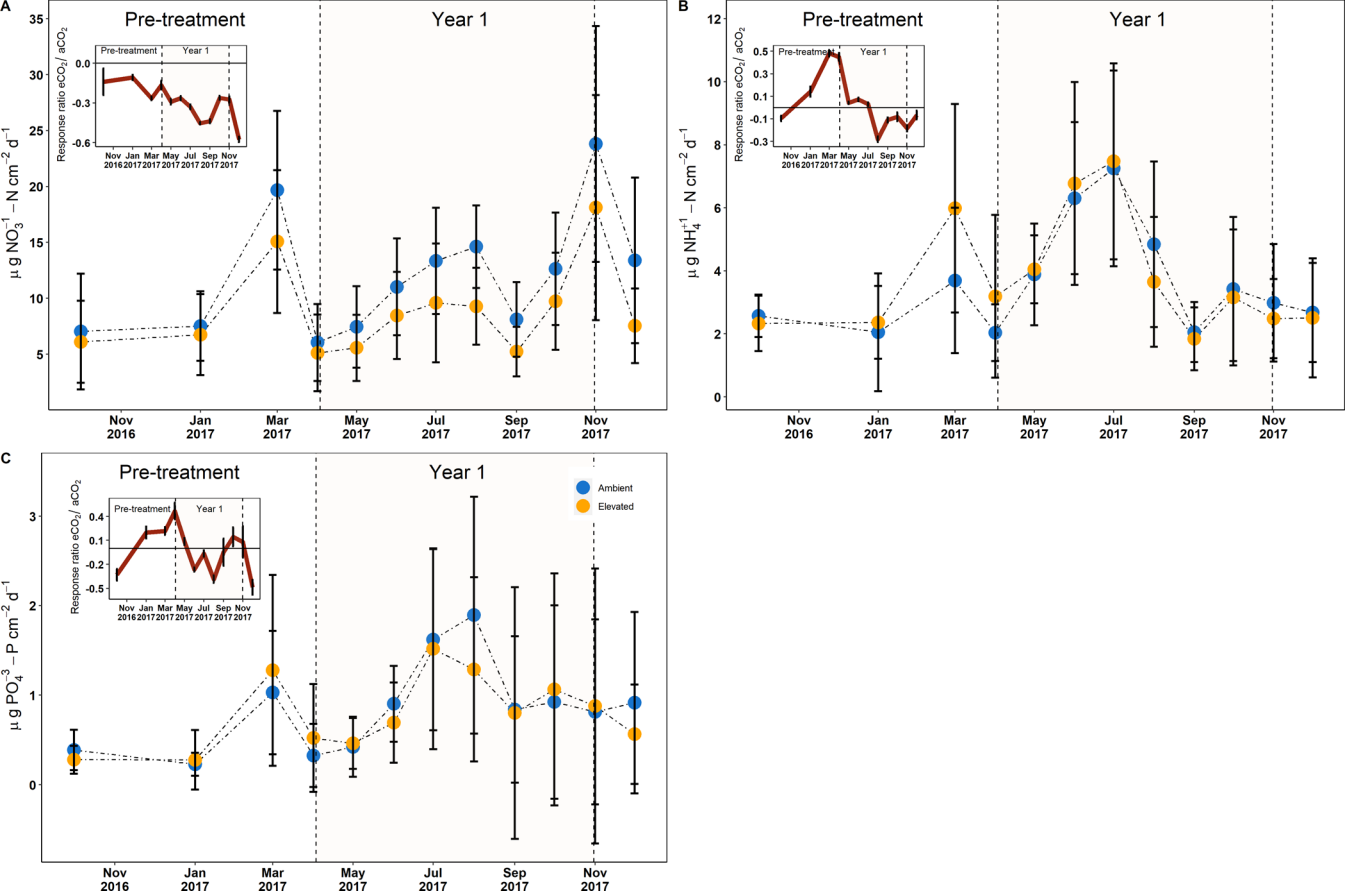
Temporal changes in ion-exchange resin membranes (a) NO_3_^–^-N, (b) NH_4_^+^-N, and (c) PO_4_^3–^-P fluxes, (mean ± sd, *n* = 3). The blue solid points reflect the nutrient availability in the aCO_2_ arrays, whereas the yellow solid points reflect the nutrient availability in the eCO_2_ arrays. The plot within each subplot represents the RR for eCO_2_/aCO_2_ and the error bars denote the standard deviation. Year 1 denotes CO_2_ enrichment (3rd April–27th October 2017), whereas the period before reflects the Pre-treatment (01 October 2016–02 April 2017).

All three bioavailable soil nutrients (NO_3_^–^-N, NH_4_^+^-N, and PO_4_^3–^-P) exhibited a strong seasonality, with availability increasing as the *T*_s_ increased and VWC decreased during Year 1 (Figure 3a–c with Figure 1b,c). Similarly, all three nutrients exhibited lower (<0) RR during the period of maximum plant productivity between June and September, indicating lower nutrient availability in eCO_2_ relative to aCO_2_ (Figure 3a–c subplots). Although the NO_3_^–^-N RR values were below zero throughout the entire study period, a sharp drop in the RR was observed to coincide with the start of eCO_2_ enrichment (dashed line in subplots). Similar trends were observed for NH_4_^+^-N, and PO_4_^3–^-P during this period of peak plant activity.

During the Pre-treatment, eCO_2_ had significantly higher NH_4_^+^-N than aCO_2_ (4.2 ± 3.1 versus 2.9 ± 2.1 μg NH_4_^+^-N cm^–2^ day^–1^; *p* = 0.027; Table 4). However, following CO_2_ enrichment, these differences were no longer apparent (3.9 ± 2.6 versus 3.8 ± 2.7 μg NH_4_^+^-N cm^–2^ day^–1^). By contrast, NO_3_^–^-N did not differ in the Pre-treatment (13.6 ± 8.5 versus 10.8 ± 6.7 μg NO_3_^–^-N cm^–2^ day^–1^) but was significantly lower under eCO_2_ following the CO_2_ enrichment start (12.3 ± 7.5 versus 8.7 ± 6.1 μg NO_3_^–^-N cm^–2^ day^–1^; *p* = 1.3 × 10^–8^). There were no differences in PO_4_^3–^-P availability in either the Pre-treatment or Year 1.

**Table 4:** Summary of mean (sd in parentheses) nutrient concentration in aCO_2_ and eCO_2_ for Pre-treatment and Year 1^a^

	Pre-treatment			Year 1		
	Mean (μg cm^–2^)			Mean (μg cm^–2^)		
Nutrient	aCO_2_	eCO_2_	*p*	aCO_2_	eCO_2_	*p*
NO_3_^–^-N	13.6 (8.5)	10.8 (6.7)	0.129	12.3 (7.5)	8.7 (6.1)	**1.3 × 10**^**–8**^
NH_4_^+^-N	2.9 (2.1)	4.2 (3.1)	**0.027**	3.9 (2.6)	3.8 (2.7)	0.631
PO_4_^3–^-P	0.64 (0.61)	0.77 (0.91)	0.649	0.97 (1.0)	0.87 (6.1)	0.383

a The parameter *p* reports the significance of the Wilcoxon Rank Sum test for differences between NO_3_^–^-N, NH_4_^+^-N, and PO_4_^3–^-P availability in aCO_2_ and eCO_2_.

**Figure 4:**
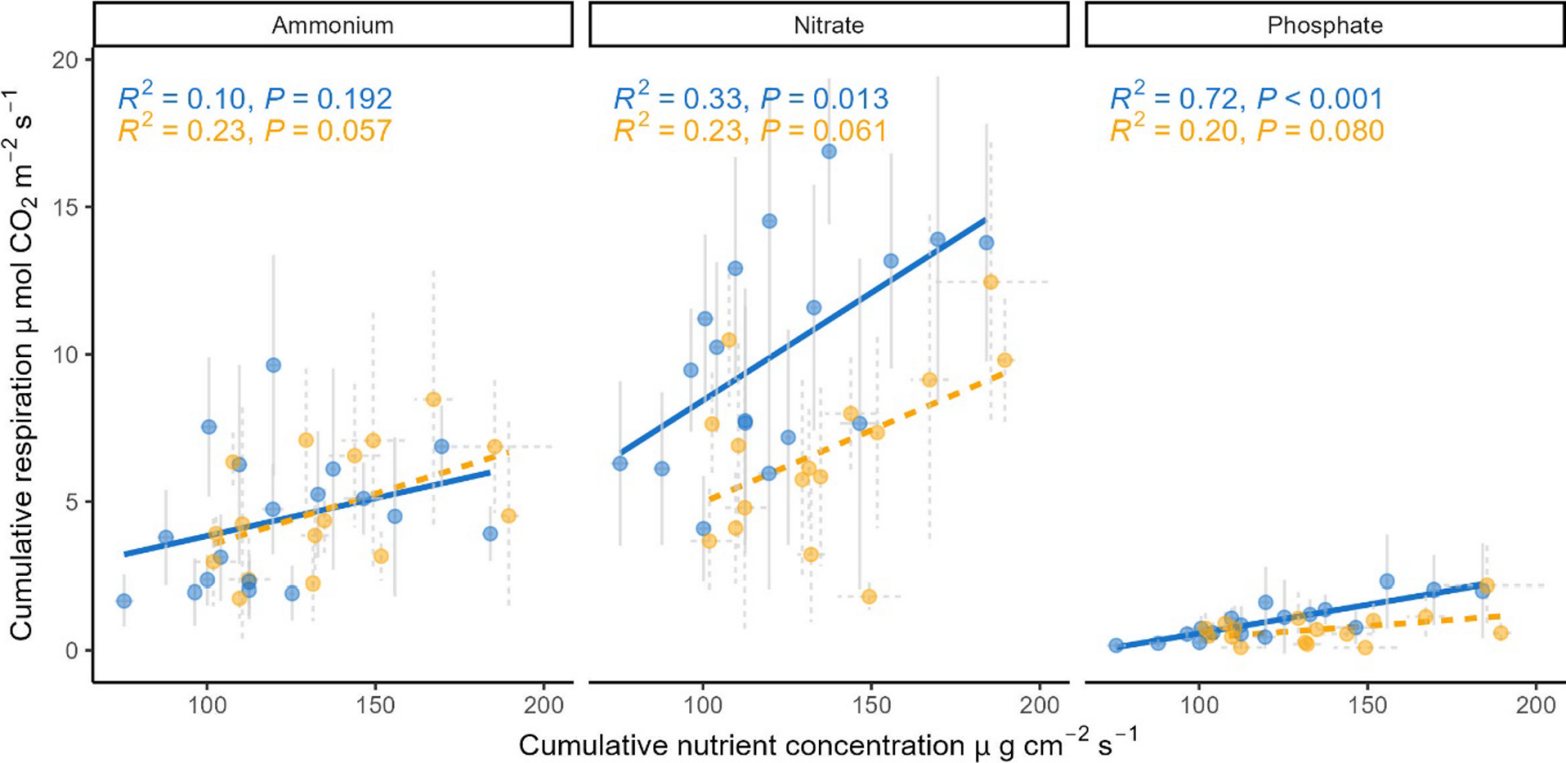
Relationships between cumulative monthly *R*_s_ and cumulative monthly nutrient concentration for NH_4_^+^-N, NO_3_^–^-N, and PO_4_^3–^-P in aCO_2_ and eCO_2_ arrays during Year 1. Blue lines, points, and text are aCO_2_, dashed yellow lines, points and text are eCO_2_. Error bars denote the standard deviation.

Cumulative monthly nutrient availability was correlated with cumulative *R*_s_ and these relationships were significant for both NO_3_^–^-N and PO_4_^3–^-P in aCO_2_ (Figure 4). Marginal significance was also apparent for all three nutrients in eCO_2_, however, they were not statistically significant, and the explained variability was low. NH_4_^+^-N was marginally significantly correlated with cumulative *R*_s_ under eCO_2_ only.

During Year 1, the RR indicated that eCO_2_ had the effect of reducing the nutrient availability in the soil. The greatest effect was during August, which coincided with some of the greatest differences in *R*_s_ between aCO_2_ and eCO_2_ (Table 2). The effect on NO_3_^–^-N availability was most pronounced with a significant decrease of up to 36%, in eCO_2_ relative to aCO_2_ (Figure 3a and Table 4). The effect of eCO_2_ on NH_4_–N^+^ and PO_4_^3–^-P availabilities was not significant, but there was an absolute reduction in P availability in eCO_2_ during Year 1.

The effect of eCO_2_ on NO_3_^–^-N availability in the literature is highly varied; it has been observed to increase, decrease or have no significant impact.^25,54,57−64^ The mechanistic pathways are difficult to trace due to the plethora of processes involved and their interactions. Dinitrogen fixation, microbial immobilization, nitrification, and denitrification rates, root uptake kinetics, and the density of roots in the soil, as well as soil structure and water availability, can all impact the soil NO_3_^–^-N availability.

The observed decrease in NO_3_^–^-N availability in eCO_2_ could be explained by a competition between plants and microbes in N uptake. Trees grown in acidic conditions (such as BIFoR FACE) prefer NH_4_^+^ disproportionately to NO_3_^–^^65^ subsequently reducing the amount of NH_4_^+^available for microbial nitrification.^54^ Decreased gross nitrification rates were observed compared to ammonification rates as well as increased denitrification under eCO_2_ at BIFoR FACE, and^30^ low nitrification rates in the rhizosphere were observed. Thus, the observed reduction in NO_3_^–^-N availability at BIFoR FACE in Year 1 was likely due to a combined low nitrification rate and increased denitrification rate combined with the preferential root uptake of NH_4_^+^.

Although more C is coming into the canopy-top leaves via increased photosynthesis, increasing leaf mass per unit area, no significant change in green leaf C:N mass fraction has been detected.^66^ This constant leaf stoichiometry suggests that N supply to trees is sustained via enhanced soil N transformation rates.^54^ Moreover, it has been observed that mature trees under eCO_2_ decreased their NO_3_^–^-N assimilation, irrespective of substrate availability,^67^ potentially assimilating more NH_4_^+^-N, further downregulating nitrification rates and subsequently decreasing NO_3_^–^-N production rates consistent with the observed decrease in soil bioavailable NO_3_^–^-N and suppressed nitrification rates in the rhizosphere.^30^ Though uptake and assimilation of both N forms are essential for plant growth, NO_3_^–^-N requires considerably more energy for uptake and assimilation than NH_4_^+^-N because NO_3_^–^-N must be metabolically reduced to ammonia before it can be assimilated.^68−71^ Thus, the distinct root preference for NH_4_^+^-N uptake is especially pronounced in tree species, possibly as an adaptation mechanism to forest soils that are relatively low in NO_3_^–^-N content,^57^ and this trait could be enhanced with higher competition for N under eCO_2_.

Although no difference was observed in NH_4_^+^-N and PO_4_^3–^-P availability between aCO_2_ and eCO_2_ during Year 1, both NH_4_^+^-N and PO_4_^3–^-P RR decreased during Year 1 relative to the Pre-treatment suggesting lower nutrient availability in eCO_2_ relative to aCO_2_. This is in contrast to other mature forests under eCO_2_ that have observed greater NH_4_^+^-N and PO_4_^3–^-P availability under eCO_2_.^25−27,66^ Plant^72,73^ as well as microbial^74^ demand for P has been reported to increase under eCO_2_ but only where its limits to productivity.^75,76^

This study focused on the first year of the CO_2_ enrichment. Continuing observation is essential since individual processes within the forest system have different time scales. Nevertheless, the high frequency and density of measurements reported above demonstrate, for the first time, the initial coupling of C and nutrient processes in a mature deciduous temperate forest under eCO_2_.

## 4 Environmental Significance

Our results, combined with previous studies at BIFoR FACE,^24,28−30,66^ show an immediate increase in C allocation belowground during the first year of CO_2_ enrichment, increasing root respiration, and the availability of respirable carbon to microorganisms. In order to meet increased metabolic demands, soil microorganisms, and plants then increased their NH_4_^+^-N uptake combined with an NH_4_^+^-N conservation due to lower nitrification rates, resulting in less available NO_3_^–^-N. The effects reported here, along with the other effects of eCO_2_ reported previously for the BIFoR FACE facility, show immediate changes in C and N biogeochemistry in this mature temperate forest with a strong seasonal cycle. However, the temperature and soil moisture interact strongly with the perturbed C and N biogeochemistry even in this mild, maritime climate, highlighting the importance of the high-frequency measurements. Although not all CO_2_ responses are long-lived if the observed responses were to be observed long-term, mature forest ecosystems with increased belowground C inputs would have to shift their nutrient-acquiring strategies or exhibit greater nutrient use efficiency to meet nutrient demands. However, further research is required in order to disentangle such complex ecosystem processes.

## Data Availability

The data that support the findings of this study are not publicly available yet but can be available on request from the Birmingham’s Institute of Forest Research (BIFoR) Data Manager.

## Supporting Information Available

Further Supporting Information for the experimental site, the measurements, and the statistical analysis; linear model parameters and evaluation of best fit and predictive quality based on *R*^2^, AIC, and RMSE of the model performance on a testing data set (PDF)

## Footnotes

1 Assuming flux difference is linearly related to soil concentration difference.

## References

[ref1] Schimel D.; Stephens B. B.; Fisher J. B. Effect of increasing CO_2_ on the terrestrial carbon cycle. Proc. Natl. Acad. Sci. U.S.A. 2015, 112 (2), 436–441. 10.1073/pnas.1407302112. 25548156 PMC4299228

[ref2] Pan Y.; Birdsey R. A.; Fang J.; Houghton R.; Kauppi P. E.; Kurz W. A.; Phillips O. L.; Shvidenko A.; Lewis S. L.; Canadell J. G.; Ciais P.; Jackson R. B.; Pacala S. W.; McGuire A. D.; Piao S.; Rautiainen A.; Sitch S.; Hayes D. A large and persistent carbon sink in the world’s forests. Science 2011, 333 (6045), 988–993. 10.1126/science.1201609. 21764754

[ref3] Le Quéré C.; Andrew R.; Friedlingstein P.; Sitch S.; Hauck J.; Pongratz J.; Pickers P.; Ivar Korsbakken J.; Peters G.; Canadell J.; Arneth A.; Arora V.; Barbero L.; Bastos A.; Bopp L.; Ciais P.; Chini L.; Ciais P.; Doney S.; Gkritzalis T.; Goll D.; Harris I.; Haverd V.; Hoffman F.; Hoppema M.; Houghton R.; Hurtt G.; Ilyina T.; Jain A.; Johannessen T.; Jones C.; Kato E.; Keeling R.; Klein Goldewijk K.; Landschützer P.; Lefèvre N.; Lienert S.; Liu Z.; Lombardozzi D.; Metzl N.; Munro D.; Nabel J.; Nakaoka S. I.; Neill C.; Olsen A.; Ono T.; Patra P.; Peregon A.; Peters W.; Peylin P.; Pfeil B.; Pierrot D.; Poulter B.; Rehder G.; Resplandy L.; Robertson E.; Rocher M.; Rödenbeck C.; Schuster U.; Skjelvan I.; Séférian R.; Skjelvan I.; Steinhoff T.; Sutton A.; Tans P.; Tian H.; Tilbrook B.; Tubiello F.; van Der Laan-Luijkx I.; van Der Werf G.; Viovy N.; Walker A.; Wiltshire A.; Wright R.; Zaehle S.; Zheng B. Global Carbon Budget 2018. Earth Syst. Sci. Data 2018, 10 (4), 2141–2194. 10.5194/essd-10-2141-2018.

[ref4] Zhao Z.; Peng C.; Yang Q.; Meng F.-R.; Song X.; Chen S.; Epule T. E.; Li P.; Zhu Q. Model prediction of biome-specific global soil respiration from 1960 to 2012. Earth’s Future 2017, 5, 715–729. 10.1002/2016EF000480.

[ref5] Sousa W. P. The Role of Disturbance in Natural Communities. Annual Review of Ecology and Systematics 1984, 15, 353–391. 10.1146/annurev.es.15.110184.002033.

[ref6] Pan Y.; Birdsey R.; Hom J.; Mccullough K. Separating effects of changes in atmospheric composition, climate and land-use on carbon sequestration of U.S. Mid-Atlantic temperate forests. Forest Ecology and Management 2009, 259 (2), 151–164. 10.1016/j.foreco.2009.09.049.

[ref7] Bonan G. B. Forests and climate change: Forcings, feedbacks, and the climate benefits of forests. Science 2008, 320 (5882), 1444–1449. 10.1126/science.1155121. 18556546

[ref8] Cook-Patton S. C.; Leavitt S. M.; Gibbs D.; Harris N. L.; Lister K.; Anderson-Teixeira K. J.; Briggs R. D.; Chazdon R. L.; Crowther T. W.; Ellis P. W.; Griscom H. P.; Herrmann V.; Holl K. D.; Houghton R. A.; Larrosa C.; Lomax G.; Lucas R.; Madsen P.; Malhi Y.; Paquette A.; Parker J. D.; Paul K.; Routh D.; Roxburgh S.; Saatchi S.; van de Hoogen J.; Walker W. S.; Wheeler C. E.; Wood S. A.; Xu L.; Griscom B. W. Mapping carbon accumulation potential from global natural forest regrowth. Nature 2020, 585, 545–550. 10.1038/s41586-020-2686-x. 32968258

[ref9] Keith H.; Mackey B. G.; Lindenmayer D. B. Re-evaluation of forest biomass carbon stocks and lessons from the world’s most carbon-dense forests. Proc. Natl. Acad. Sci. U.S.A. 2009, 106 (28), 11635–11640. 10.1073/pnas.0901970106. 19553199 PMC2701447

[ref10] Zaehle S.; Medlyn B. E.; De Kauwe M. G.; Walker A. P.; Dietze M. C.; Hickler T.; Luo Y.; Wang Y.-P.; El-Masri B.; Thornton P.; Jain A.; Wang S.; Warlind D.; Weng E.; Parton W.; Iversen C. M.; Gallet-Budynek A.; McCarthy H.; Finzi A.; Hanson P. J.; Prentice I. C.; Oren R.; Norby R. J. Evaluation of 11 terrestrial carbon-nitrogen cycle models against observations from two temperate Free-Air CO_2_ enrichment studies. New Phytologist 2014, 202, 803–822. 10.1111/nph.12697. 24467623 PMC4288990

[ref11] Körner C. Plant CO_2_ responses: an issue of definition, time and resource supply. New Phytologist 2006, 172, 393–411. 10.1111/j.1469-8137.2006.01886.x. 17083672

[ref12] Körner C.; Asshoff R.; Bignucolo O.; Hättenschwiler S.; Keel S. G.; Peláez-Riedl S.; Pepin S.; Siegwolf R. T. W.; Zotz G. Carbon flux and growth in mature deciduous forest trees exposed to elevated CO_2_. Science 2005, 309 (5739), 1360–1362. 10.1126/science.1113977. 16123297

[ref13] Sitch S.; Huntingford C.; Gedney N.; Levy P. E.; Lomas M.; Piao S. L.; Betts R.; Ciais P.; Cox P.; Friedlingstein P.; Jones C. D.; Prentice I. C.; Woodward F. I. Evaluation of the terrestrial carbon cycle, future plant geography and climate-carbon cycle feedbacks using five Dynamic Global Vegetation Models (DGVMs). Global Change Biology 2008, 14 (9), 2015–2039. 10.1111/j.1365-2486.2008.01626.x.

[ref14] Anderson-Teixeira K. J.; Heermann V.; Morgan R. B.; Bond-Lamberty B.; Cook-Patton S. C.; Ferson A. E.; Muller-Landau H. C.; Wang M. M. H. Carbon cycling in mature and regrowth forests globally. Environ. Res. Lett. 2021, 16 (5), 053009 10.1088/1748-9326/abed01.

[ref15] Hawkes C. V.; Waring B. G.; Rocca J. D.; Kivlin S. N. Historical climate controls soil respiration responses to current soil moisture. Proc. Natl. Acad. Sci. U.S.A. 2017, 114 (24), 6322–6327. 10.1073/pnas.1620811114. 28559315 PMC5474806

[ref16] Kuzyakov Y.; Horwath W. R.; Dorodnikov M.; Blagodatskaya E. Review and synthesis of the effects of elevated atmospheric CO_2_ on soil processes: No changes in pools but increased fluxes and accelerated cycles. Soil Biology and Biochemistry 2019, 128, 66–78. 10.1016/j.soilbio.2018.10.005.

[ref17] Ellsworth D. S.; Anderson I. C.; Crous K. Y.; Cooke J.; Drake J. E.; Gherlenda A. N.; Gimeno T. E.; Macdonald C. A.; Medlyn B. E.; Powell J. R.; Tjoelker M. G.; Reich P. B. Elevated CO_2_ does not increase eucalypt forest productivity on a low-phosphorus soil. Nature Climate Change 2017, 7, 279–282. 10.1038/nclimate3235.

[ref18] Terrer C.; Jackson R. B.; Prentice I. C.; Keenan T. F.; Kaiser C.; Vicca S.; Fisher J. B.; Reich P. B.; Stocker B. D.; Hungate B. A.; Penuelas J.; McCallum I.; Soudzilovskaia N. A.; Cernusak L. A.; Talhelm A. F.; van Sundert K.; Piao S.; Newton P. C. D.; Hoveden M. J.; Blumental D. M.; Liu Y. Y.; Muller C.; Winter K.; Field C. B.; Viechtbauer W.; Van Lissa C. J.; Hoosbeek M. R.; Watanabe M.; Koike T.; Leshyk V. O.; Polley H. W.; Franklin O. Nitrogen and phosphorus constrain the CO_2_ fertilization of global plant biomass. Nat. Clim. Chang. 2019, 9, 684–689. 10.1038/s41558-019-0545-2.

[ref19] Du E.; Terrer C.; Pellegrini A. F. A.; Ahlstrom A.; van Lissa C. J.; Zhao X.; Xia N.; Wu X.; Jackson R. B. Global patterns of terrestrial nitrogen and phosphorus limitation. Nature Geoscience 2020, 13, 221–226. 10.1038/s41561-019-0530-4.

[ref20] Jiang M.; Crous K. Y.; Carrillo Y.; Macdonald C. A.; Anderson I. C.; Boer M. M.; Farrell M.; Gherlenda A. N.; Castañeda-Gómez L.; Hasegawa S.; Jarosch K.; Milham P. J.; Ochoa-Hueso R.; Pathare V.; Pihlblad J.; Piñeiro J.; Powell J. R.; Power S. A.; Reich P. B.; Riegler M.; Zaehle S.; Smith B.; Medlyn B. E.; Ellsworth D. S. Microbial competition for phosphorus limits the CO_2_ response of a mature forest. Nature 2024, 630, 660–665. 10.1038/s41586-024-07491-0. 38839955 PMC11186757

[ref21] King J. S.; Hanson P. J.; Bernhardt E. S.; DeAngelis P.; Norby R. J.; Pregitzer K. S. A multiyear synthesis of soil respiration response to elevated atmospheric CO_2_ from four forest FACE experiments. Glob. Chang. Biol. 2004, 10, 1027–1042. 10.1111/j.1365-2486.2004.00789.x.

[ref22] Reich P. B.; Hungate B. A.; Luo Y. Carbon-Nitrogen Interactions in Terrestrial Ecosystems in Response to Rising Atmospheric Carbon Dioxide. Annual Review of Ecology, Evolution, and Systematics 2006, 37, 611–636. 10.1146/annurev.ecolsys.37.091305.110039.

[ref23] Reich P. B.; Hobbie S. E. Decade-long soil nitrogen constraint on the CO_2_ fertilization of plant biomass. Nature Climate Change 2013, 3, 278–282. 10.1038/nclimate1694.

[ref24] Norby R. J.; Loader N. J.; Mayoral C.; Ullah S.; Curioni G.; Smith A. R.; Reay M. K.; van Wijngaarden K.; Amjad M. S.; Brettle D.; Crockatt M. E.; Denny G.; Grzesik R. T.; Hamilton R. L.; Hart K. M.; Hartley I. P.; Jones A. J.; Kourmouli A.; Larsen J. R.; Shi Z.; Thomas R. M.; MacKenzie A. R. Enhanced woody biomass production in a mature temperate forest under elevated CO_2_. Nature Climate Change 2024, 14, 983–988. 10.1038/s41558-024-02090-3.

[ref25] Hasegawa S.; Macdonald C. A.; Power S. A. Elevated carbon dioxide increases soil nitrogen and phosphorus availability in a phosphorus-limited Eucalyptus woodland. Global Change Biology 2016, 22, 1628–1643. 10.1111/gcb.13147. 26546164

[ref26] Ochoa-Hueso R.; Hughes J.; Delgado-Baquerizo M.; Drake J. E.; Tjoelker M. G.; Piñeiro J.; Power S. A. Rhizosphere-driven increase in nitrogen and phosphorus availability under elevated atmospheric CO_2_ in a mature Eucalyptus woodland. Plant and Soil 2017, 416, 283–295. 10.1007/s11104-017-3212-2.

[ref27] Pihlblad J.; Andersen L. C.; Macdonald C. A.; Ellsworth D. S.; Carrillo Y. The influence of elevated CO_2_ and soil depth on rhizosphere activity and nutrient availability in a mature Eucalyptus woodland. Biogeosciences 2023, 20 (3), 505–521. 10.5194/bg-20-505-2023.

[ref28] Gardner A.; Ellsworth D.; Crous K.; Pritchard J.; Mackenzie A. R. Is photosynthetic enhancement sustained through three years of elevated CO2 exposure in 175-year-old Quercus robur?. Tree Physiology 2022, 42 (1), 130–144. 10.1093/treephys/tpab090. 34302175 PMC8754963

[ref29] Ziegler C.; Kulawska A.; Kourmouli A.; Hamilton L.; Shi Z.; MacKenzie A. R.; Dyson R. J.; Johnston I. G. Quantification and uncertainty of root growth stimulation by elevated CO_2_ in mature temperate deciduous forest. Sci. Total Environ. 2023, 854, 158661 10.1016/j.scitotenv.2022.158661. 36096230

[ref30] Rumeau M.; Sgouridis F.; MacKenzie A. R.; Carrillo Y.; Reay M. K.; Hartley I. P.; Ullah S. The role of rhizosphere in enhancing N availability in a mature temperate forest under elevated CO_2_. Soil Biol. Biochem. 2024, 197, 109537 10.1016/j.soilbio.2024.109537.

[ref31] Hollis J.; Jones B.; Ullah S.; MacKenzie A. R.; Curioni G.; Hart K. Soil Profile Pit at BIFoR-FACE, Norbury Junction, Staffordshire; University of Birmingham, 2021.

[ref32] Hart K. M.; Curioni G.; Blaen P.; Harper N. J.; Miles P.; Lewin K. F.; Nagy J.; Bannister E. J.; Cai X. M.; Thomas R. M.; Krause S.; Tausz M.; MacKenzie A. R. Characteristics of free air carbon dioxide enrichment of a northern temperate mature forest. Glob. Chang. Biol. 2020, 26 (2), 1023–1037. 10.1111/gcb.14786. 31376229 PMC7027798

[ref33] MacKenzie R.; Krause S.; Hart K.; Thomas R.; Blaen P.; Hamilton R.; Curioni G.; Quick S.; Kourmouli A.; Hannah D.; Comer-Warner S.; Brekenfeld N.; Ullah S.; Press M. BIFoR FACE: Water-soil-vegetation-atmosphere research in a temperate deciduous forest catchment, including under elevated CO_2_. Hydrol. Process. 2021, 35 (3), e14096 10.22541/au.160157598.86879557.

[ref34] Crowley L. M.; Sadler J. P.; Pritchard J.; Hayward S. A. L.; Mccabe M.; Pitts-Singer T.; Koch J. B. Elevated CO2 Impacts on Plant–Pollinator Interactions: A Systematic Review and Free Air Carbon Enrichment Field Study. Insects 2021, 12, 512 10.3390/INSECTS12060512. 34206033 PMC8227562

[ref35] Norby R. J.; De Kauwe M. G.; Domingues T. F.; Duursma R. A.; Ellsworth D. S.; Goll D. S.; Lapola D. M.; Luus K. A.; Mackenzie A. R.; Medlyn B. E.; Pavlick R.; Rammig A.; Smith B.; Thomas R.; Thonicke K.; Walker A. P.; Yang X.; Zaehle S. Model-data synthesis for the next generation of forest free-air CO_2_ enrichment (FACE) experiments. New Phytologist 2016, 209, 17–28. 10.1111/nph.13593. 26249015

[ref36] Arias P. A.; Bellouin N.; Coppola E.; Jones R. G.; Krinner G.; Marotzke J.; Naik V.; Palmer M. D.; Plattner G.-K.; Rogelj J.; Rojas M.; Sillmann J.; Storelvmo T.; Thorne P. W.; Trewin B.; Achuta Rao K.; Adhikary B.; Allan R. P.; Armour K.; Bala G.; Barimalala R.; Berger S.; Canadell J. G.; Cassou C.; Cherchi A.; Collins W.; Collins W. D.; Connors S. L.; Corti S.; Cruz F.; Dentener F. J.; Dereczynski C.; Di Luca A.; Diongue Niang A.; Doblas-Reyes F. J.; Dosio A.; Douville H.; Engelbrecht F.; Eyring V.; Fischer E.; Forster P.; Fox-Kemper B.; Fuglestvedt J. S.; Fyfe J. C.; Gillett N. P.; Goldfarb L.; Gorodetskaya I.; Gutierrez J. M.; Hamdi R.; Hawkins E.; Hewitt H. T.; Hope P.; Islam A. S.; Jones C.; Kaufman D. S.; Kopp R. E.; Kosaka Y.; Kossin J.; Krakovska S.; Lee J.-Y.; Li J.; Mauritsen T.; Maycock T. K.; Meinshausen M.; Min S.-K.; Monteiro P. M. S.; Ngo-Duc T.; Otto F.; Pinto I.; Pirani A.; Raghavan K.; Ranasinghe R.; Ruane A. C.; Ruiz L.; Sallée J.-B.; Samset B. H.; Sathyendranath S.; Seneviratne S. I.; Sörensson A. A.; Szopa S.; Takayabu I.; Tréguier A.-M.; van den Hurk B.; Vautard R.; von Schuckmann K.; Zaehle S.; Zhang X.; Zickfeld K.; Technical Summary. In Climate Change 2021: The Physical Science Basis. Contribution of Working Group I to the Sixth Assessment Report of the Intergovernmental Panel on Climate Change. Masson-Delmotte V.; Zhai P.; Pirani A.; Connors S.L.; Péan C.; Berger S.; Caud N.; Chen Y.; Goldfarb L.; Gomis M.I.; Huang M.; Leitzell K.; Lonnoy E.; Matthews J.B.R.; Maycock T.K.; Waterfield T.; Yelekçi O.; Yu R.; Zhou B. (eds.).; Cambridge University Press: Cambridge, United Kingdom and New York, NY, USA, 2021; 33–144.

[ref37] Rayment G.; Lyons D. J. Soil chemical methods: Australasia (Australian soil and land survey handbook series: v.3); CSIRO Publishing, 2010.

[ref38] Bowatte S.; Tillman R.; Carran A.; Gillingham A.; Scotter D. In situ ion exchange resin membrane (IEM) technique to measure soil mineral nitrogen dynamics in grazed pastures. Biology and Fertility of Soils 2008, 44, 805–813. 10.1007/s00374-007-0260-4.

[ref39] Worsfold P. J.; Gimbert L. J.; Mankasingh U.; Omaka O. N.; Hanrahan G.; Gardolinski P. C. F. C.; Haygarth P. M.; Turner B. L.; Keith-Roach M. J.; McKelvie I. D. Sampling, sample treatment and quality assurance issues for the determination of phosphorus species in natural waters and soils. Talanta 2005, 66 (2), 273–293. 10.1016/j.talanta.2004.09.006. 18969993

[ref40] Hedges L. V.; Gurevitch J.; Curtis P. S. The meta-analysis of response ratios in experimental ecology. Ecological Society of America 1999, 80 (4), 1150–1156. 10.1890/0012-9658(1999)080[1150:TMAORR]2.0.CO;2.

[ref41] Rstrudio Team. RStudio: Integrated Development for R. 2019.

[ref42] Bond-Lamberty B.; Pennington S. C.; Jian J.; Megonigal J. P.; Sengupta A.; Ward N. Soil Respiration Variability and Correlation Across a Wide Range of Temporal Scales. JGR Biogeosciences 2019, 124 (11), 3672–3683. 10.1029/2019JG005265.

[ref43] Bates D.; Mächler M.; Bolker B.; Walker S. Fitting Linear Mixed-Effects Models Using lme4. Journal of Statistical Software 2015, 67 ( (1), ). 10.18637/jss.v067.i01.

[ref44] Barton K. MuMIn: multi-model inference. R package version 3.1–96. 2014.

[ref45] Fox J.; Weisberg S. An R Companion to Applied Regression, 3rd ed.; Sage: Thousand Oaks CA, 2019.

[ref46] Akaike H. Information theory and an extension of the maximum likelihood principle. In: Petrov B. N.; Csáki F, editors. 2nd International Symposium on Information Theory; 1971 Sep 2–8; Tsahkadsor, Armenia, USSR; Akadémiai Kiadó: Budapest, 1973, 267–281.

[ref47] Campbell J. L.; Law B. E. Forest soil respiration across three climatically distinct chronosequences in Oregon. Biogeochemistry 2005, 73, 109–125. 10.1007/s10533-004-5165-9.

[ref48] Xu M.; Shang H. Contribution of soil respiration to the global carbon equation. Journal of Plant Physiology 2016, 203 (20), 16–28. 10.1016/j.jplph.2016.08.007. 27615687

[ref49] Jiang M.; Medlyn B. E.; Drake J. E.; Duursma R. A.; Anderson I. C.; Barton C. V. M.; Boer M. M.; Carrillo Y.; Castañeda-Gómez L.; Collins L.; Crous K. Y.; De Kauwe M. G.; Dos Santos B. M.; Emmerson K. M.; Facey S. L.; Gherlenda A. N.; Gimeno T. E.; Hasegawa S.; Johnson S. N.; Kännaste A.; Macdonald C. A.; Mahmud K.; Moore B. D.; Nazaries L.; Neilson E. H. J.; Nielsen U. N.; Niinemets Ü.; Noh N. J.; Ochoa-Hueso R.; Pathare V. S.; Pendall E.; Pihlblad J.; Piñeiro J.; Powell J. R.; Power S. A.; Reich P. B.; Renchon A. A.; Riegler M.; Rinnan R.; Rymer P. D.; Salomón R. L.; Singh B. K.; Smith B.; Tjoelker M. G.; Walker J. K. M.; Wujeska-Klause A.; Yang J.; Zaehle S.; Ellsworth D. S. The fate of carbon in a mature forest under carbon dioxide enrichment. Nature 2020, 580, 227–231. 10.1038/s41586-020-2128-9. 32269351

[ref50] Drake J. E.; Macdonald C. A.; Tjoelker M. G.; Crous K. Y.; Gimeno T. E.; Singh B. K.; Reich P. B.; Anderson I. C.; Ellsworth D. S. Short-term carbon cycling responses of a mature eucalypt woodland to gradual stepwise enrichment of atmospheric CO_2_ concentration. Global Change Biology 2016, 22 (1), 380–390. 10.1111/gcb.13109. 26426394

[ref51] Drake J. E.; Macdonald C. A.; Tjoelker M. G.; Reich P. B.; Singh B. K.; Anderson I. C.; Ellsworth D. S. Three years of soil respiration in a mature eucalypt woodland exposed to atmospheric CO_2_ enrichment. Biogeochemistry 2018, 139, 85–101. 10.1007/s10533-018-0457-7.

[ref52] Giardina C. P.; Ryan M. G. Total belowground carbon allocation in a fast-growing Eucalyptus plantation estimated using a carbon balance approach. Ecosystems 2002, 5, 487–499. 10.1007/s10021-002-0130-8.

[ref53] Drake J. E.; Gallet-Budynek A.; Hofmockel K. S.; Bernhardt E. S.; Billings S. A.; Jackson R. B.; Johnsen K. S.; Lichter J.; Mccarthy H. R.; Mccormack M. L.; Moore D. J. P.; Oren R.; Palmroth S.; Phillips R. P.; Pippen J. S.; Pritchard S. G.; Treseder K. K.; Schlesinger W. H.; Delucia E. H.; Finzi A. C. Increases in the flux of carbon belowground stimulate nitrogen uptake and sustain the long-term enhancement of forest productivity under elevated CO_2_. Ecology Letters 2011, 14 (4), 349–357. 10.1111/j.1461-0248.2011.01593.x. 21303437

[ref54] Sgouridis F.; Reay M.; Cotchim S.; Ma J.; Radu M.; Ullah S. Stimulation of soil gross nitrogen transformations and nitrous oxide emission under Free air CO_2_ enrichment in a mature temperate oak forest at BIFoR-FACE. Soil Biol. Biochem. 2023, 184, 109072 10.1016/j.soilbio.2023.109072.

[ref55] Kuzyakov Y. Priming effects: Interactions between living and dead organic matter. Soil Biology and Biochemistry 2010, 42 (9), 1363–1371. 10.1016/j.soilbio.2010.04.003.

[ref56] van Groenigen K. J.; Qi X.; Osenberg C. W.; Luo Y.; Hungate B. A. Faster decomposition under increased atmospheric CO_2_ limits soil carbon storage. Science 2014, 344, 508–509. 10.1126/science.1249534. 24762538

[ref57] Bassirirad H.; Thomas R. B.; Reynolds J. F.; Strain B. R. Differential responses of root uptake kinetics of NH_4_^+^ and NO_3_^–^ to enriched atmospheric CO_2_ concentration in field-grown loblolly pine. Plant, Cell and Environment 1996, 19, 367–371. 10.1111/j.1365-3040.1996.tb00260.x.

[ref58] Bassirirad H.; Griffin K. L.; Reynolds J. F.; Strain B. R. Changes in root NH_4_^+^ and NO_3_^–^ absorption rates of loblolly and ponderosa pine in response to CO_2_ enrichment. Plant and Soil 1997, 190, 1–9. 10.1023/A:1004206624311.

[ref59] Hungate B. A.; Lund C. P.; Pearson H. L.; Chapin F. S. Elevated CO_2_ and nutrient addition after soil N cycling and N trace gas fluxes with early season wet-up in a California annual grassland. Biogeochemistry 1997, 37, 89–109. 10.1023/A:1005747123463.

[ref60] Niklaus P. A.; Kandeler E.; Leadley P. W.; Schmid B.; Tscherko D.; Korner C. A link between plant diversity, elevated CO_2_ and soil nitrate. Oecologia 2001, 127 (4), 540–548. 10.1007/s004420000612. 28547492

[ref61] Luo Y.; Su B.; Currie W. S.; Dukes J. S.; Finzi A. C.; Hartwig U.; Hungate B.; McMurtrie R. E.; Oren R.; Parton W. J.; Pataki D. E.; Shaw M. R.; Zak D. R.; Field C. B. Progressive nitrogen limitation of ecosystem responses to rising atmospheric carbon dioxide. BioScience 2004, 54 (8), 731–739. 10.1641/0006-3568(2004)054[0731:PNLOER]2.0.CO;2.

[ref62] Hagedorn F.; Maurer S.; Bucher J. B.; Siegwolf R. T. W. Immobilization, stabilization and remobilization of nitrogen in forest soils at elevated CO_2_: a ^15^N and ^13^C tracer study. Global Change Biology 2005, 11 (10), 1816–1827. 10.1111/j.1365-2486.2005.01041.x.

[ref63] Schleppi P.; Bucher-Wallin I.; Hagedorn F.; Körner C. Increased nitrate availability in the soil of a mixed mature temperate forest subjected to elevated CO_2_ concentration (canopy FACE). Global Change Biology 2012, 18, 757–768. 10.1111/j.1365-2486.2011.02559.x.

[ref64] Schleppi P.; Körner C.; Klein T. Increased Nitrogen Availability in the Soil Under Mature Picea abies Trees Exposed to Elevated CO_2_ Concentrations. Frontiers in Forests and Global Change 2019, 2, 2 10.3389/ffgc.2019.00059.

[ref65] Zhang Y.; Bai S. Effects of nitrogen forms on nutrient uptake and growth of trees. Ying Yong Sheng Tai Xue Bao 2003, 14 (11), 2044–2048. 14997674

[ref66] Gardner A.; Ellsworth D. S.; Pritchard J.; MacKenzie A. R. Are chlorophyll concentrations and nitrogen across the vertical canopy profile affected by elevated CO_2_ in mature Quercus trees?. Trees 2022, 36, 1797–1809. 10.1007/s00468-022-02328-7.

[ref67] Wujeska-Klause A.; Crous K. Y.; Ghannoum O.; Ellsworth D. S. Lower photorespiration in elevated CO_2_ reduces leaf N concentrations in mature Eucalyptus trees in the field. Global Change Biology 2019, 25 (4), 1282–1295. 10.1111/gcb.14555. 30788883

[ref68] Haynes R. J.; Goh K. M. Ammonium and nitrate nutrition of plants. Biological Reviews 1978, 53, 465–510. 10.1111/j.1469-185X.1978.tb00862.x.

[ref69] Blacquière T.; Hofstra R.; Stulen I. Ammonium and nitrate nutrition in *Plantago lanceolata* and *Plantago major L. ssp. major -* I. Aspects of growth, chemical composition and root respiration. Plant and Soil 1987, 104 (1), 129–141. 10.1007/BF02370635.

[ref70] Glass A. D. M.; Siddiqi M. Y. Nitrogen absorption by plant roots, in: Srivastava H. S.; Singh R. P. (Eds.), Nitrogen Nutrition in Higher Plants; Associated Pub. Co: New Delhi, 1995.

[ref71] Maathuis F. J. Physiological functions of mineral macronutrients. Current Opinion in Plant Biology 2009, 12 (3), 250–258. 10.1016/j.pbi.2009.04.003. 19473870

[ref72] Gentile R.; Dodd M.; Lieffering M.; Brock S. C.; Theobald P. W.; Newton P. C. D. Effects of long-term exposure to enriched CO_2_ on the nutrient-supplying capacity of a grassland soil. Biology and Fertility of Soils 2012, 48, 357–362. 10.1007/s00374-011-0616-7.

[ref73] Zhang C.; Niu D.; Hall S. J.; Wen H.; Li X.; Fu H.; Wan C.; Elser J. J. Effects of simulated nitrogen deposition on soil respiration components and their temperature sensitivities in a semiarid grassland. Soil Biology and Biochemistry 2014, 75, 113–123. 10.1016/j.soilbio.2014.04.013.

[ref74] Keane J. B.; Hartley I. P.; Taylor C. R.; Leake J. R.; Hoosbeek M. R.; Miglietta F.; Phoenix G. K. Grassland responses to elevated CO_2_ determined by plant-microbe competition for phosphorus. Nat. Geosci. 2023, 16, 704–709. 10.1038/s41561-023-01225-z.

[ref75] Johnson D. W.; Cheng W.; Joslin J. D.; Norby R. J.; Edwards N. T.; Todd D. E. Effects of elevated CO_2_ on nutrient cycling in a sweetgum plantation. Biogeochemistry 2004, 69, 379–403. 10.1023/B:BIOG.0000031054.19158.7c.

[ref76] Dijkstra F. A.; Pendall E.; Morgan J. A.; Blumenthal D. M.; Carrillo Y.; Lecain D. R.; Follett R. F.; Williams D. G. Climate change alters stoichiometry of phosphorus and nitrogen in a semiarid grassland. New Phytologist 2012, 196 (3), 807–815. 10.1111/j.1469-8137.2012.04349.x. 23005343

