# Supplementary material for: Rapid Increase in Soil Respiration and Reduction in Soil Nitrate Availability Following CO2 Enrichment in a Mature Oak Forest
Source: ACS Omega. 2025 Jan 2;10(1):1624–34. doi: 10.1021/acsomega.4c09495 (PMC11739980; doi:10.1021/acsomega.4c09495)
Supplement: Supplementary file 1 — ao4c09495_si_001.pdf [file ao4c09495_si_001.pdf]

Rapid increase in soil respiration and reduction in soil nitrate availability following CO_2 enrichment in a mature oak forest

Angeliki Kourmouli[1,2*]; R. Liz Hamilton[1,3]; Johanna Pihlblad[1,2]; Rebecca Bartlett[1,3]; Angus Robert MacKenzie[1,3]; Iain P. Hartley[4]; Sami Ullah[1,3].; Zongbo Shi[1,3]

1. Birmingham Institute of Forest Research (BIFoR), University of Birmingham, Edgbaston, B15 2TT, United Kingdom

2. Lancaster Environment Centre, Lancaster University, Bailrigg, LA1 4YQ, United Kingdom

3. School of Geography, Earth & Environmental Sciences, University of Birmingham, Edgbaston, B15 2TT, United Kingdom

4. Geography, Faculty of Environment, Science and Economy, University of Exeter, Exeter, EX4 4RJ, United Kingdom

*Correspondence to email: a.kourmouli@lancaster.ac.uk;

Supporting Information

Supporting Methodology

Experimental site

The enrichment system operates from just before dawn (solar zenith angle, sza = -5o, ascending) to just after sunset (sza = -5o, descending). Contamination of the aCO_2 arrays by CO_2 from the eCO_2 arrays only occurred during 1.2% of the operational time and was still within the 10% of the control set point.

Measurements

Soil respiration R_s

Automated measurements of R_s were taken simultaneously at 1-hour intervals at all three surface collars in a fumigated array and its paired ambient control array. The observation length was 2 min with 20 s dead-band, a 15 s pre-purge, and a 45 s post-purge, giving a total measurement cycle of 3 min 20 s.

Soil respiration automated equipment designed for long term deployment will sometimes fail due to extreme conditions and external factors, and respiration measurements are inherently highly variable, however we are confident that our continuous maintenance and calibration, based on manufacturer recommendations, and data quality assurance processes mean the data included in this study is of high quality. Data with poor quality fits, including negative linear fluxes and those linear fluxes derived from observations when the coefficient of variation is higher than 3.5 were removed. Such data are indicative of leaks in the chamber. Data points with a linear flux coefficient of variation between 1.5 and 3.5 were manually checked to assess the fit quality. There was a moderate data loss (43%), primarily due to equipment failures (water condensation in the electric plates) and, to a lesser extent, due to poor-quality fits.

Figure S1 shows the number of measurements per day from the 19th October 2016 until the 31st December 2017 in aCO_2 and eCO_2 arrays, respectively (total number of days 438). The aCO_2 arrays were measured in total for 258 days (41.1% loss) while the eCO_2 were measured for 341 days (22.1% loss). The longest consecutive periods without measurements were 19 days for aCO_2 and 22 days for eCO_2. While this is not optimal, it is worth noting that there were still 11 and 8 days with measurements during the impacted months, respectively. Of the days that were measured, 90.3% of the measured days had data loss less than 20% for aCO_2 arrays, while 84.5% of the measured days had data loss less than 20% for eCO_2 arrays.

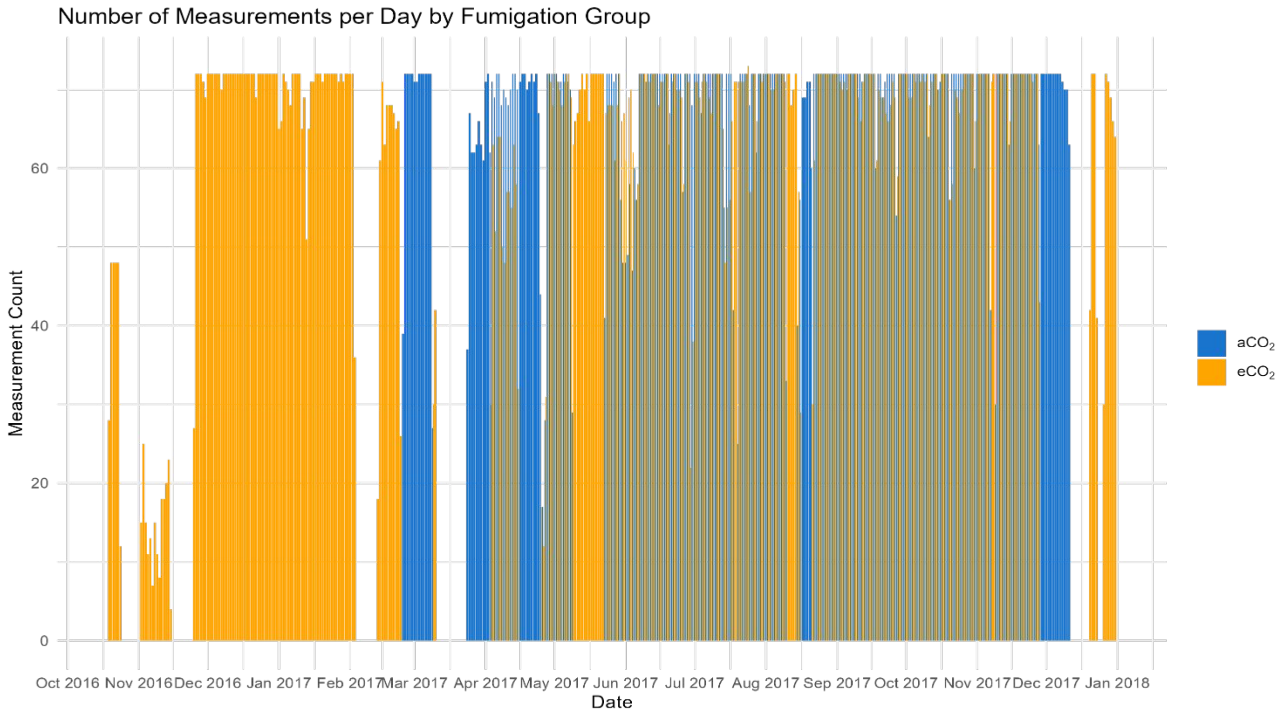

Figure S1: Number of measurements per day from the 19th of October 2016 until the 31st of December 2017 in the aCO_2 (blue) and eCO_2 (yellow) arrays, respectively.

Biota-available (bioavailable) inorganic N and P

Ion-exchange resin membranes were cut into 2 x 12 cm strips. A hole was punched at the top 2 cm to allow for polypropylene string to be thread through, allowing for easier identification while deployed in the field. The cation-exchange resin membranes were saturated with H^+ by shaking the strips overnight with 2 M HCl, while the anion-exchange resin membranes were

saturated with HCO_3^- by shaking the strips overninght with 1 M $NaHCO_3$. Both resin membranes were rinsed free of excess HCl and $NaHCO_3$ with deionised water and left in deionised water for another day with frequent change of the water to remove the last trace of acid.

To assess the resin membranes' loading capacity, a capacity lab test was performed for 60 days, testing daily (data not shown). Up to 40 days of resin membrane incubation, 99% of NH_4^+-N, NO_3^--N, and PO_4^{3-}-P was absorbed in the membrane; beyond 40 days, the absorbance capacity decreased, but never to less than 90%.

Following the linear regression approach (Shrivastava and Gupta, 2011) for calculating the quality of the analytical methods, the limits of detection were 0.01 and 0.004 mg/L for NH_4^+ and NO_3^- respectively, the limits of quantitation were 0.03 and 0.01 mg/L for NH_4^+ and NO_3^- respectively. Following the same approach for PO_4^{3-}, the limit of detection was 0.0005 mg/L, the limit of quantitation was 0.002mg/L.

Statistical analysis

Environmental drivers and R_s

Treatment effects were derived directly by comparing the daily means of R_s for eCO_2 and ambient arrays using mixed effects models. The Pre-treatment period and Year 1 were assessed separately to establish potential significant differences before eCO_2 enrichment and significant treatment effects, respectively. Interactions between CO_2 treatment, Time, and environmental drivers (VWC and T_s) were evaluated using mixed effects models for both periods. In all mixed-effects models performed, ‘array,’ and ‘number of days since the beginning of the experiment’ to account for the correlations between repeated measurements within each array were added as random effects. In all mixed-effects models performed in this study daily averages of R_s (per collar), VWC, and T_s (per sensor, respectively) were used.

Mixed-effects models were used to investigate potential pre-eCO_2 enrichment differences in the pre-assigned as 'ambient' and 'elevated' arrays. Although there was no eCO_2 enrichment during the Pre-treatment period, for consistency with subsequent data the arrays are named 'ambient' and 'elevated' throughout. The CO_2 treatment, Time, VWC and T_s, as well as their interactions were treated as fixed effects. The two models with the lowest AIC score were compared using the Anova function to compute the Chi-squares between the two models for investigating the model fit. Finally, the coefficient of determination (R^2) was calculated to assess the variance captured by the model. Each model was checked for homoscedasticity, normality, linearity of residuals, and linearity of the random effects.

The same principles were used for investigating eCO_2 effects during Year 1 of eCO_2 enrichment. The CO_2 treatment, Time, VWC and T_s, as well as their interactions (CO_2 treatment*Time, CO_2 treatment*Time*VWC, CO_2 treatment*Time*T_s and CO_2 treatment*Time*VWC*T_s) were treated as fixed effects.

Bioavailable nutrients

For nutrient analysis, the replication unit was the FACE array (n=3 for each of ambient and eCO_2), and all data were aggregated into monthly data per array. Data collected before the eCO_2 enrichment were analysed separately to assess initial pre-treatment differences. Time was assessed as a fixed factor but had no effect on all three soil nutrients availabilities. In this mixed-effects framework, 'array' and 'number of days since the beginning of the measurements' were used as random factors. Subsequently, mixed-effects models were undertaken with CO_2 treatment and two covariates – T_s and VWC – and their interactions (CO_2 treatment*T_s and CO_2 treatment*VWC) as fixed effects. Similarly, the same pattern was followed during Year 1 to evaluate both the potential eCO_2 effect as well to evaluate the role of soil conditions in responses to elevated CO_2.

Estimation of annual R_s

Table S1. Linear model parameters and evaluation of best fit and predictive quality based on r2, AIC, and RMSE of the model performance on a testing data set. Row in bold is best model fit for ambient and elevated model respectively and used to predict annual R_s. R_s was log transformed to fullfill model assumptions.

	Slope	*intercept*	*p*	*Adj. R^2*	AIC	RMSE
Ambient						
$R_s = \square_0 + VWC + \varepsilon$	-0.04143	1.531944	0	0.3058	3657.43	1.17
$R_s = \square_0 + T_s + \varepsilon$	0.097667	-0.03212	0	0.5755	1588.07	0.95
**$R_s = \square_0 + VWC{*}\ T_s + \varepsilon$**	**-0.02814**	**0.407938**	**0**	**0.5882**	**1462.66**	**0.93**
Elevated						
$R_s = \square_0 + VWC + \varepsilon$	-0.04171	1.784209	0	0.3097	4841.78	1.43
$R_s = \square_0 + T_s + \varepsilon$	0.09531	0.149323	0	0.6224	2065.67	1.09
**$R_s = \square_0 + VWC{*}\ T_s + \varepsilon$**	**-0.03536**	**0.843306**	**0**	**0.6519**	**1692.39**	**1.03**
